# Clinical, biochemical and molecular analysis in a cohort of individuals with gyrate atrophy

**DOI:** 10.1186/s13023-023-02840-0

**Published:** 2023-09-04

**Authors:** Eleanor Palmer, Karolina M. Stepien, Christopher Campbell, Stephanie Barton, Christos Iosifidis, Arunabha Ghosh, Alexander Broomfield, Alison Woodall, Gisela Wilcox, Panagiotis I. Sergouniotis, Graeme C. Black

**Affiliations:** 1 Manchester Royal Eye Hospital, Manchester University NHS Foundation Trust, Manchester, UK; 2 Adult Inherited Metabolic Disorders, Salford Royal NHS Foundation Trust, Salford, Greater Manchester UK; 3 Division of Diabetes, Endocrinology and Gastroenterology, School of Medical Sciences, Faculty of Biology, Medicine and Health, University of Manchester, Manchester, UK; 4 Manchester Centre for Genomic Medicine, Saint Mary’s Hospital, Manchester University NHS Foundation Trust, Manchester, UK; 5 Willink Biochemical Genetics, Royal Manchester Children’s Hospital, Manchester University NHS Foundation Trust, Manchester, UK; 6 Division of Evolution, Infection and Genomics, School of Biological Sciences, Faculty of Biology, Medicine and Health, University of Manchester, Manchester, UK

**Keywords:** Gyrate atrophy, Inborn errors of metabolism, Inherited retinal disorders, Hyperornithinaemia, Ornithine aminotransferase deficiency, OAT

## Abstract

**Background:**

Gyrate atrophy of the choroid and retina is a rare autosomal recessive metabolic disorder caused by biallelic variants in the *OAT* gene, encoding the enzyme ornithine δ-aminotransferase. Impaired enzymatic activity leads to systemic hyperornithinaemia, which in turn underlies progressive chorioretinal degeneration. In this study, we describe the clinical and molecular findings in a cohort of individuals with gyrate atrophy.

**Methods:**

Study participants were recruited through a tertiary UK clinical ophthalmic genetic service. All cases had a biochemical and molecular diagnosis of gyrate atrophy. Retrospective phenotypic and biochemical data were collected using electronic healthcare records.

**Results:**

18 affected individuals from 12 families (8 male, 10 female) met the study inclusion criteria. The median age at diagnosis was 8 years (range 10 months – 33 years) and all cases had hyperornithinaemia (median: 800 micromoles/L; range: 458–1244 micromoles/L). Common features at presentation included high myopia (10/18) and nyctalopia (5/18). Ophthalmic findings were present in all study participants who were above the age of 6 years. One third of patients had co-existing macular oedema and two thirds developed pre-senile cataracts. Compliance with dietary modifications was suboptimal in most cases. A subset of participants had extraocular features including a trend towards reduced fat-free mass and developmental delay.

**Conclusions:**

Our findings highlight the importance of multidisciplinary care in families with gyrate atrophy. Secondary ophthalmic complications such as macular oedema and cataract formation are common. Management of affected individuals remains challenging due to the highly restrictive nature of the recommended diet and the limited evidence-base for current strategies.

**Supplementary Information:**

The online version contains supplementary material available at 10.1186/s13023-023-02840-0.

## Background

Gyrate atrophy of the choroid and retina (also known as ornithine δ-aminotransferase deficiency) is an inborn error of metabolism primarily affecting the eye. This rare condition is inherited as an autosomal recessive trait and is associated with biallelic pathogenic variants in the *OAT* gene. *OAT* encodes ornithine aminotransferase, a vitamin B6-dependent enzyme that catalyses the conversion of the urea cycle substrate ornithine to pyrroline 5-carboxylate, which can be subsequently converted into the amino acids glutamate and proline [1, 2].

Over 80 pathogenic variants have been described in *OAT* including nonsense, frameshift, splice site and, most commonly, missense variants. These changes lead to a significant reduction in enzymatic activity resulting in the accumulation of ornithine in the plasma (hyperornithinaemia), urine and cerebrospinal fluid of affected individuals [3, 4]. Notably, high ornithine concentrations are associated with damage to retinal tissue and over time lead to progressive visual loss [5–10].

Affected individuals usually present in the first or second decade of life, commonly with night vision problems and/or progressive myopia. [5, 6, 10, 11]. Following this, concentric visual field loss is generally noted during the second decade of life. This slowly progresses to severe loss of vision at around the fourth to fifth decade [5–8, 10, 11]. Loss of visual acuity may be secondary to macular oedema and/or photoreceptor cell loss. Early lens opacification is common and most affected individuals require cataract surgery at a relatively young age [12–15].

The retinal manifestations of gyrate atrophy are highly characteristic and strongly point towards the diagnosis. Fundus examination shows sharply demarcated, scalloped areas of chorioretinal atrophy arising peripherally in the retina. These are initially patchy but gradually enlarge, become confluent and spread centrally towards the macula. Non-ophthalmic manifestations have been described including neonatal hyperammonaemia, intellectual disability, reduced muscle function and bone disorders; some of these findings point to potential long-term consequences of high ornithine levels on other pathways particularly creatine and lysine metabolism [5, 6, 8, 10, 11].

There is presently no cure for gyrate atrophy and treatment options remain limited. A low-protein diet with synthetic amino acid supplementation is recommended and has been demonstrated to reduce the rate of retinal degeneration in an animal model [9]. These dietary modifications aim to reduce plasma ornithine levels and to slow disease progression [7–10, 16, 17]. However, compliance with diet is often suboptimal, particularly in adolescents and adults, reflecting the difficulties in adjusting to a low protein diet particularly if this is initiated after infancy or early childhood.

Here, we report clinical and genetic findings in 18 individuals with gyrate atrophy. Retinal and systemic manifestations are discussed and the importance of close multi-disciplinary management is highlighted.

## Methods

### Study participants

Study subjects were retrospectively ascertained through the database of the North West Genomic Laboratory Hub, Manchester, UK. Only families who were diagnosed through the tertiary clinics at Manchester University NHS Foundation Trust and Salford Royal NHS Foundation Trust, Manchester, UK were included. Study participants were referred for genetic testing between 2017 and 2020 and had a homozygous or (at least) two heterozygous pathogenic or likely pathogenic variants in *OAT*. Segregation analysis was not performed in all cases.

Ethics committee approval for the study was obtained from the North West Research Ethics Committee (11/NW/0421 and 15/YH/0365) and all investigations were conducted in accordance with the tenets of the Declaration of Helsinki.

### Phenotypic data collection & clinical genetic testing

The clinical notes and/or electronic healthcare record entries were reviewed for each study participant. A full clinical history was obtained and most study subjects were assessed by an ophthalmologist, a metabolic physician and a geneticist. A subset of participants underwent fundus imaging, including widefield retinal imaging, fundus autofluorescence imaging, optical coherence tomography (OCT), and OCT-angiography (OCT-A). The Optos system (Optos PLC, Dunfermline, Scotland, UK) was used to obtain widefield images, the Topcon DRI OCT Triton device (Topcon GB, Newberry, Berkshire, UK) was used to obtain OCT and OCT-A scans, and the Spectralis system (Heidelberg Engineering, Heidelberg, Germany) was used to acquire fundus autofluorescence and OCT images in study participants.

Blood samples were obtained from all probands and DNA was extracted. Multigene panel testing and analysis were subsequently performed at the North West Genomic Laboratory Hub (ISO 15189:2012; UKAS Medical reference 9865) using a previously described approach [18].

## Results

### Clinical and imaging findings

Overall, 18 patients (8 male, 10 female) from 12 families met the study inclusion criteria. This included 15 adults and 3 paediatric patients. Clinical, biochemical and genetic findings in this cohort are shown in Table 1 (See Table 1 in Additional File 1). The majority of study participants (n = 17) had south-east Asian ancestries.

**Table 1:** Molecular diagnosis, clinical and biochemical findings in a cohort of 18 patients with gyrate atrophy

Patient No	Gender	Age Range at Last Ophthalmic Examination (years)	Visual Acuity (LogMAR)		Presenting Ophthalmic Symptom(s) (Age at Diagnosis [years])	Presence of Macular Oedema (Y/N)	Presence of Cataracts (Y/N)	Plasma Ornithine Levels at Diagnosis (μmol/L) [NR: 40–150 µmol/L]	Average Plasma Ornithine in Last 5 Years (μmol/L)	Average Plasma Lysine in Last 5 Years (μmol/L) [NR: 100–160 μmol/L]	Genetic Diagnosis, Pathogenic/Likely Pathogenic Variant
			Right eye	Left eye							
1	M	31–35	0.4	1.0	Increasing myopia (14)	Y	Y	1120	1067	87	c.520 + 1G > A (homozygous)
2	M	21–25	0.68	0.54	Increasing myopia (9)	Y	Y	1018	1342	73	c.520 + 1G > A (homozygous)
3	F	26–30	0.22	0.24	Increasing myopia (11)	N	Y	1007	863	72	c.520 + 1G > A (homozygous)
4	M	15–20	0.32	0.44	Asymptomatic (2)	Y	N	458	868	84	p.(Arg398Ter) (homozygous)
5	F	26–30	0.42	0.3	Asymptomatic (5)	Y	Y	695	564	87	p.(Arg398Ter) (homozygous)
6	F	21–25	0.34	0.34	Increasing myopia (6)	N	Y	1244	N/A	N/A	p.(Arg398Ter) (homozygous)
7	F	5–14	0.1	0.1	Increasing myopia (8)	N	N	576	528	150	p.(Pro241Leu) (homozygous)
8	M	5–14	0.38	0.3	Asymptomatic (4)	N	N	754	597	130	p.(Pro241Leu) (homozygous)
9	F	21–25	0.5	0.64	Nyctalopia, Reduced peripheral vision (8)	Y	N	775	640	106	p.(Pro241Leu) (homozygous)
10	F	31–35	0.94	0.9	Increasing myopia, Nyctalopia, Reduced peripheral vision (7)	N	Y	826	572	76	p.(Pro241Leu) (homozygous)
11	F	26–30	0.5	0.6	Increasing myopia, Nyctalopia, Cataracts (26)	Y	Y	800	718	204	p.(Pro241Leu) (homozygous)
12	F	26–30	0.3	0.4	Increasing myopia, Nyctalopia (14)	N	Y	734	641	103	p.(Gly51Asp) (homozygous)
13	M	21–25	0.16	0.3	Asymptomatic (0.83)	N	Y	458	392	169	p.(Arg398Ter) (homozygous)
14	M	0–4	0.0	0.0	Asymptomatic (1)	N	N	1232	879	195	p.(Pro241Leu); (heterozygous) p.(Gly353Asp) (heterozygous)
15	F	21–25	0.22	0.4	Increasing myopia (14)	N	Y	742	716	108	p.(Arg250Ter); (heterozygous) p.(Ile314Ser) (heterozygous)
16	F	41–45	0.0	-0.1	Increasing myopia, Nyctalopia, Cataracts (33)	N	Y	917	N/A	N/A	p.(Tyr209Ter); (heterozygous) p.(Pro417Leu) (heterozygous)
17	M	15–20	0.22	0.5	Increasing myopia, Floaters (14)	N	N	N/A	458	247	p.(Leu403Pro); (heterozygous) (Leu337ArgfsTer2) (heterozygous)
18	M	26–30	0.48	0.6	Asymptomatic (5)	N	Y	920	N/A	N/A	p.(Pro300LeufsTer13) (homozygous)

NB: Two patients for whom genetic data only were available have been excluded from the above table and information regarding their genetic diagnosis is included in Table 4

*Y* = yes, *N* = no

The median age at diagnosis was 8 years (range: 10 months – 33 years). All affected individuals had plasma ornithine levels above the normal range at the time of diagnosis (range: 458 – 1244 µmol/L) (reference range: 40–150 µmol/L, provided by the Willink Metabolic Laboratory, which was responsible for analysis of all samples referenced in this study). Common features at presentation included myopia (10/18 cases) and night vision problems (5/18 cases). Ophthalmic symptoms were present in all study participants who were above the age of 6 years (age at which either myopia or nyctalopia became apparent). The median age when chorioretinal changes were first clinically documented was 13 years (range: 4–33 years). The median visual acuity at the most recent ophthalmic assessment was 0.36 LogMAR units (range: 0.0–0.7 LogMAR). One third of study participants had non-leaking macular oedema for which a course of treatment with carbonic anhydrase inhibitors was offered including oral acetazolamide in most (5/6) cases. Notably, 4 of the 6 patients affected by macular oedema were also noted to develop pre-senile cataracts. Overall, 12 of the 18 study participants (66.6%) developed pre-senile cataracts in early adulthood; 7 of these cases underwent cataract surgery with the median age of intervention for this subset being 27.5 years (range: 22–36 years). Five affected individuals were clinically documented as sight impaired registered, though limited data were available to confirm registration for most patients. Other ophthalmic features included epiretinal membrane (n = 3), floaters (n = 2), optic nerve drusen (n = 1), chronic anterior uveitis (n = 2), amblyopia (n = 1) and exophoria (n = 1).

The median body mass index (BMI) for the adult subset of the cohort (15/18) was 23.4 kg/m^2^. Six participants were found to have learning difficulties (33%) and 13 study subjects (72%) had other extraocular features including those listed in Table 2. (See Table 2 in Additional File 1).

**Table 2:** Comorbidities observed in 13 individuals with gyrate atrophy

Organ system	Comorbidity	Number of patients affected
Psychological/Neurological	Developmental delay	6
	Depression	2
	Psychosis	1
	Anorexia nervosa	1
	Dyslexia	1
	Dyspraxia	1
	Spastic dysplegia	1
	Reduced fat-free mass	9
Haematological	Anaemia (unspecified)	1
	Congenital dyserythropoeitic anaemia	1
	Red cell degeneration	1
Respiratory	Asthma	2
	Tuberculosis	1
	Vocal cord nodules	1
Cardiovascular	Congenital heart disease	1
Endocrinological	Precocious puberty	1
	Diabetes insipidus	1
Urological	Dysfunctional bladder syndrome	1

**Fig. 1:**
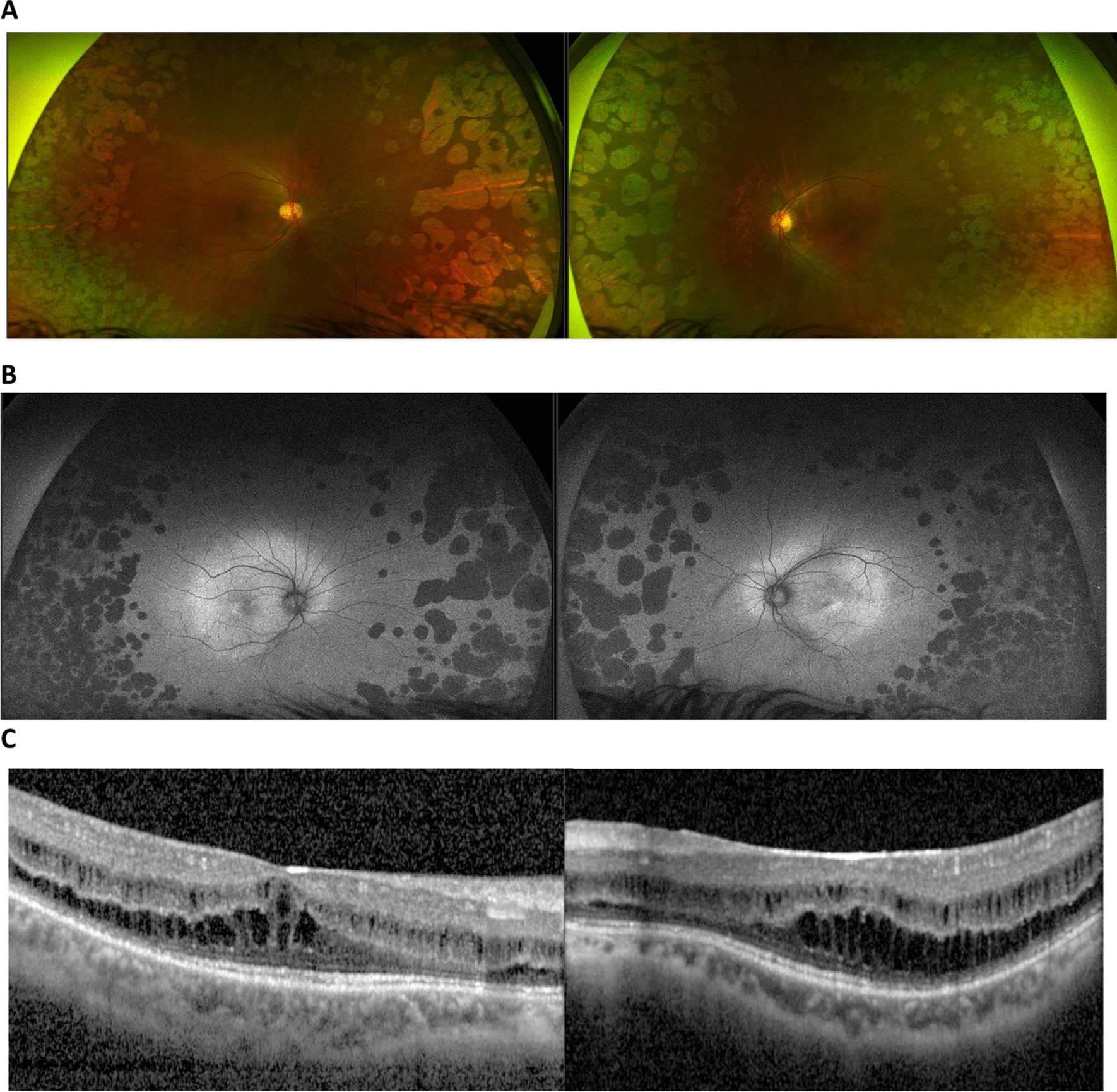
Clinical imaging findings from a 22-year-old patient with gyrate atrophy (case 2). **A** Colour fundus images demonstrating characteristic bilateral scalloped, peripheral lesions; **B** Fundus autofluorescence (FA) imaging demonstrating patchy hypo-autofluorescent lesions peripherally in keeping with RPE atrophy; **C** Optical coherence tomography (OCT) images of the macula demonstrating bilateral cystoid macular oedema, a known complication of gyrate atrophy

**Fig. 2:**
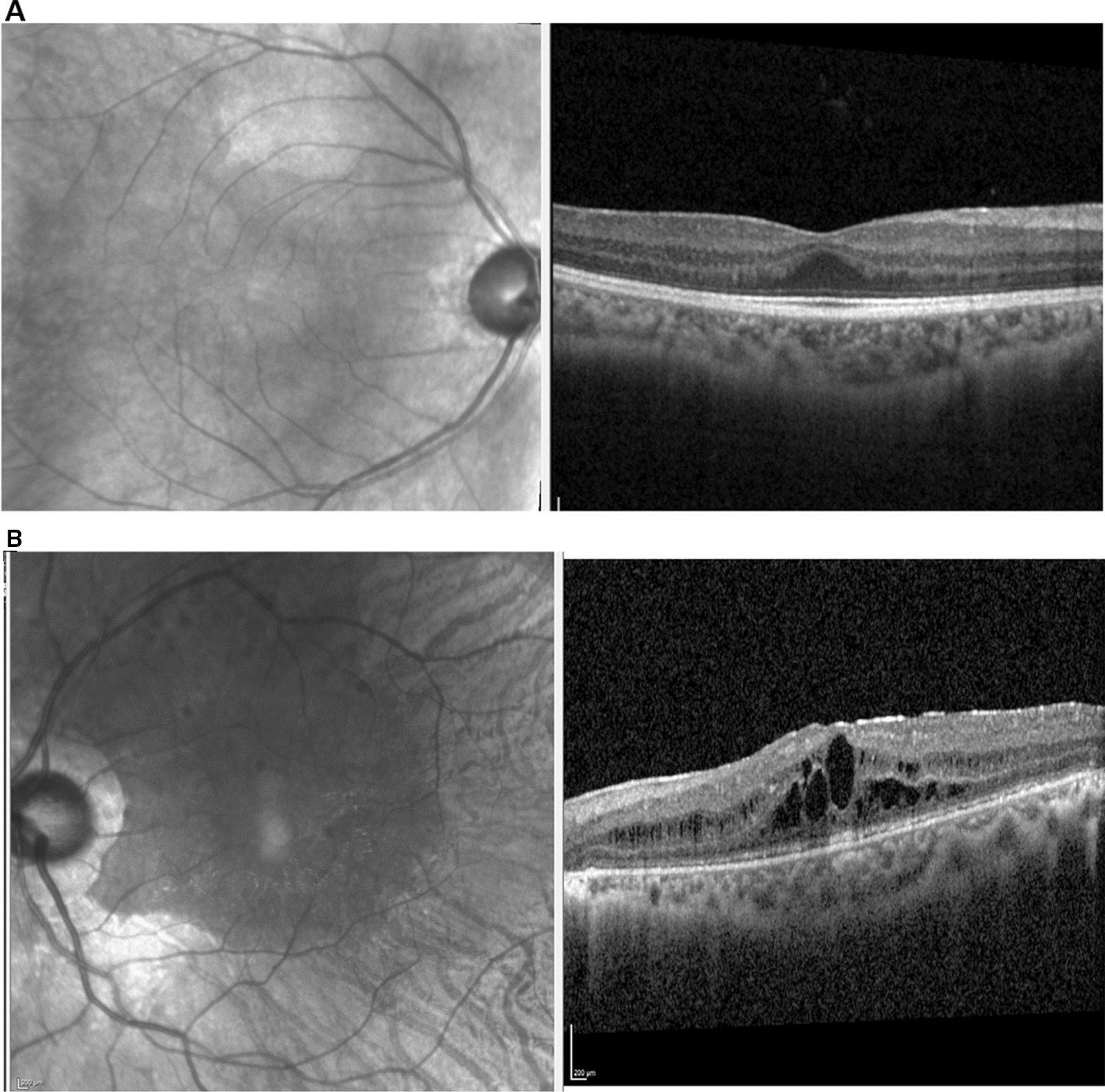
Infrared reflectance and OCT images from two unrelated study subjects with gyrate atrophy. Images from the right eye of case 13 (at age 23 years; **A**) and from the left eye of case (at 22 years; **B**) are shown. Case 13 was diagnosed in infancy following an initial presentation with hyperammonaemia, followed a strict dietary protein restriction from time of diagnosis and had what can be described as an unusually mild phenotype (visual acuity 0.16 LogMAR right and 0.3 LogMAR left). Case 2 was 9 years of age at time of diagnosis, was non-compliant with the recommended diet and went on to develop ophthalmic sequelae including cataract and cystoid macular oedema formation (visual acuity 0.68 LogMAR right and 0.54 LogMAR left). The relevant genetic diagnoses are shown in Table 4

Retinal imaging data were reviewed. Figure 1 (See Fig. 1) shows the clinical imaging findings (colour fundus imaging, fundus autofluorescence and OCT) in one patient with gyrate atrophy to illustrate the classical fundus appearance. Figure 2 (See Fig. 2) shows a visual comparison using clinical imaging from two patients with different phenotypes, highlighting that gyrate atrophy can have variable expressivity, with poorly understood genotype/phenotype correlations. Five study participants were not imaged in the 5 years preceding data collection, one of whom was an infant. Due to the SARS-CoV-2 pandemic, the provision of imaging was found to be reduced. Over the past 5 years, the group of patients who were imaged received some form of imaging at a median of 8 times (mean: 9.5 times, range: once to 22 times). With regards to OCT imaging, individuals in this subset had imaging at a median value of four times in the past 5 years (mean: 4.1, range: 7 months to > 60 months). OCT-A imaging was not frequently performed in this cohort as only two patients were imaged using this modality in the past 5 years. Fundus autofluorescence and widefield retinal images were obtained on average once every 12 months (median 12: range 5 months to > 60 months).

### Biochemical findings

Biochemical data are presented in Table 3 (See Table 3 in Additional File 1). Plasma ornithine was measured at each clinic appointment typically every 6 months. The median plasma ornithine level at diagnosis was 800 µmol/L (range: 458–1244 µmol/L). The data on fasting status prior to the sample collection were not available. As stated previously the median age at time of diagnosis was 8 years (range: 10 months – 33 years). The cohort was divided into those participants diagnosed by the age of 5 years or less (n = 6), and those diagnosed at age 6 years or older (n = 12). The 6 individuals who were diagnosed at age 5 years or younger had a median plasma ornithine of 719 µmol/L (mean: 753, range: 458–1232). The median plasma ornithine over the last 5 years for this subset was 581 µmol/L (mean: 626, range: 392–879), representing a 19% decrease. Of the 12 patients diagnosed above age 6 years, 9 had complete data for plasma ornithine levels at the time of diagnosis and in the last 5 years prior to data collection. Those diagnosed after the age of 6 years had a median plasma ornithine of 800 µmol/L at diagnosis (mean: 844, range 576–1120) and 716 µmol/L (mean: 787, range 528–1342), representing an 11% decrease.

**Table 3:** Biochemical and visual outcomes at most recent clinic visit using current management strategies

Patient no	Compliance with current treatment (Y/N/P)§	Protein restriction (daily intake [g/kg] excluding EAA)	Daily Lysine Supplementation (g)	Current additional amino acid supplement regime with total daily dosage (where available)	Plasma ornithine at diagnosis (NR: 40–150 μmol/L)	Average plasma ornithine in last 5 years (μmol/L)	Average plasma lysine in last 5 years (NR: 100–160 μmol/L)	Average visual acuity at last ophthalmic examination (logMAR)
1	P	No restriction	5	None	1120	1067	87	0.7
2	N	No restriction	0	None	1018	1342	73	0.61
3	P	0.5–0.6	0	None	1007	863	72	0.23
4	Y	1	4	EAA (3–4 sachets)	458	868	84	0.38
5	P	No restriction	4	EAA (3–4 sachets)	695	564	87	0.36
7	Y	0.5–0.8	0	EAA (4 sachets) UCD Amino 5 (1 sachet) Dialamine (100 g)	576	528	150	0.1
8	Y	0.2–0.3	0	EAA (4 sachets)	754	597	130	0.34
9	Y	1	4	None	775	640	106	0.57
10	Y	0.7–1	10	None	826	572	76	0.92
11	P	No restriction		EAA Pyridoxine (300 mg)	800	718	204	0.55
12	P	No restriction	10	EAA (4 sachets)	734	641	103	0.35
13	Y	0.15	0	Dialamine (22 scoops) Phlexyvits Zinc biotin	458	392	169	0.23
14	Y	0.4	0	EAA (3 sachets) Docomega	1232	879	195	0.0
15	N	No restriction	4	None	742	716	108	0.31
17	Y	0.5–0.6	0	Dialamine (100 g) Fruityvit	+	458	247	0.36

Cases 7, 8 and 14 are paediatric patients; cases 6, 16 and 18 had no available recorded data on ornithine or lysine levels

^§^ Y/P/N: Yes = compliant with both protein restriction, Partial = compliant with supplementation but not protein restriction, N = not compliant. Compliance was determined by patient metabolic medicine/dietician appointments

+ Data not available

At the time of diagnosis, all study participants were advised to follow a low protein diet with additional essential amino acid supplementation in an attempt to lower plasma ornithine. For adults, the recommended target for natural protein, depending on the individual, was between 0.6 and 0.8 g/kg/day. If protein intake was reported as less than 0.8 g/kg/day, essential amino acid supplementation was recommended with the aim of achieving a total protein intake of 1 g/kg/day. Other supplements included pyridoxine (in case of pyridoxine responsive OAT deficiency), lysine (to competitively reduce renal tubular reabsorption of ornithine), and creatine (to correct the creatine deficiency resulting from inhibition of synthesis by elevated plasma ornithine) [15]. Irrespective of their genotype, all newly diagnosed patients had a trial of pyridoxine, at a dose of at least 200-400 mg, taken daily for a minimum of 2 weeks. Over the past 5 years, the median plasma ornithine for the whole cohort was 678.5 µmol/L (range: 392–1342 µmol/L), representing an overall percentage decrease of 15% compared to the plasma ornithine at the time of diagnosis.

Information on dietary compliance was collected for 15 patients within the cohort. Six patients did not restrict dietary protein at all (cases 1, 2, 5, 11, 12, and 15). This subset had a median plasma ornithine at time of diagnosis and over the past 5 years of 771 µmol/L (mean: 852 µmol/L) and 717 µmol/L (mean: 841 µmol/L) respectively, representing a 7% decrease. The median visual acuity at the time of last ophthalmic examination for participants who did not restrict protein intake was 0.45 logMAR (range: 0.31 – 0.7 logMAR) at a median patient age of 26 years. For those individuals who did restrict protein intake and for whom complete data was available (n = 8), the median plasma ornithine at time of diagnosis, and over the past 5 years, was 764.5 µmol/L (mean: 761 µmol/L) and 597 µmol/L (mean: 668 µmol/L) respectively, representing a 22% decrease. For this subset who did limit dietary protein, the median visual acuity recorded at last ophthalmic examination was 0.34 logMAR (range, 0.0 – 0.92 logMAR) at a median age of 21 years. No significant difference was observed when comparing change in visual acuity between the diet restricted and non-diet restricted groups from time of diagnosis to 5 years follow up. Similarly, no significant difference was observed between groups with respect to change in plasma ornithine from time of diagnosis to 5 years follow up. When assessed as separate cohorts, serial visual acuity was reviewed for 7 study subjects; 4 of these could be classified as not-diet restricted cases while the remaining 3 would be considered diet restricted cases. A statistically significant decrease in visual acuity was noted in the former group (p = 0.025) with respect to visual acuity at diagnosis and visual acuity at 11 years following diagnosis. For the same time period, no significant difference was observed in the diet-restricted group with respect to change in visual acuity.

The most frequently utilised amino acid supplement within the cohort (n = 7) was Essential Amino Acids (EAA) (Vitaflo International Ltd, Liverpool, UK) at a dosage of between 3–4 sachets per day on average. Cases 10, 11, 12, 13 took a vitamin D supplement in addition to their current daily supplement regime (2000 units daily). Cases 1 and 10 took an additional folic acid supplement. Case 10 also took a ferrous sulphate supplement. Cases 1, 5, 13 and 15 took additional creatinine monohydrate peptide supplementation. The median plasma lysine over the past 5 years in the rest of the cohort was 124 µmol/L (range 72- 247 µmol/L). 6 out of 15 patients had plasma lysine < 100 µmol/L (normal range 100–260 µmol/L) and 7 patients required supplementation (lysine 4 g/day). Two study participants (cases 2 and 4) saw an increase in plasma ornithine from the time at diagnosis compared with the overall average over the past 5 years; this was probably related to their poor dietetic compliance (see Table 3 in Additional File 1).

### Genetic findings

Overall, 14 different likely disease-causing DNA variants in *OAT* were detected. Five of these changes have not been previously reported (See Table 4 in Additional File 1). The variants identified in the cohort include missense (n = 7), nonsense (n = 3), frameshift (n = 2) and splicing (n = 2) variants.

**Table 4:** Genetic Variants and in silico analysis

HGVS description (NM_000274.4)	REVEL score	GnomAD (v2.1.1) Number of non-ref alleles / total number of alleles	Reference (Clinvar & HGMD)
c.520 + 1G > A	n/a	Absent	Not reported on HGMD
c.461G > A p.(Arg154His)	0.929	2/251370	Ghosh et al. 2017
c.1192C > T p.(Arg398Ter)	n/a	2/250954	Michaud et al. 1995
c.722C > T p.(Pro241Leu)	0.8999	9/251486	Brody et al. 1992
c.152G > A p.(Gly51Asp)	0.875	3/251488	Sergouniotis et al. 2012
c.648G > C p.?	n/a	Absent	Not reported on HGMD
c.899delC p.(Pro300LeufsTer13)	n/a	Absent	Patel et al. 2018
c.1058 G > A p.(Gly353Asp)	0.9219	10/250560	Brody et al. 1992
c.748C > T p.(Arg250Ter)	n/a	2/251456	Sergouniotis et al. 2012
c.941 T > G p.(Ile314Ser)	0.9169	Absent	Not reported on HGMD
c.627 T > A p.(Tyr209Ter)	n/a	6/282864	Mashima et al. 1992
c.1250C > T p.(Pro417Leu)	0.8889	8/282378	Brody et al. 1992
c.1208 T > C p.(Leu403Pro)	0.9449	2/282564	Not reported on HGMD
c.1009dup p.(Leu337ArgfsTer2)	n/a	Absent	Not reported on HGMD

Ghosh et al. https://pubmed.ncbi.nlm.nih.gov/28468868/

Michaud et al. https://pubmed.ncbi.nlm.nih.gov/1612597/

Brody et al. https://pubmed.ncbi.nlm.nih.gov/1737786/

Sergouniotis et al. https://pubmed.ncbi.nlm.nih.gov/22182799/

Patel et al. https://pubmed.ncbi.nlm.nih.gov/30054919/

Mashima et al. https://pubmed.ncbi.nlm.nih.gov/1609808/

Of the previously unreported variants identified in this study, *OAT* c.1009dupC p.(Leu337ArgfsTer2) is a pathogenic frameshift change in exon 8 of 10 of the gene, which is predicted to elicit nonsense-mediated decay. This variant was identified in a heterozygous state with another novel heterozygous missense variant, c.1208 T > C p.(Leu403Pro), in case 17. This missense change affects a highly conserved leucine residue and in silico evidence predicts that a proline substitution at this position will have a damaging effect. Notably, this is the only participant in the cohort who has European ancestries. The phase of the two variants has not been established for this case, however the same genotype has been detected in an unrelated proband, in whom compound heterozygosity was proven by parental testing (unpublished data).

Another previously unreported variant is c.520 + 1G > A. This change affects the canonical splice donor site in intron 4 of the *OAT* gene and is predicted to cause in-frame skipping of exon 4. This likely pathogenic variant is absent from the “controls/biobanks” subset of the Genome Aggregation Database (gnomAD v.2.1.1) dataset and is present in the homozygous state in the three affected members of a family in this cohort. Another novel variant, *OAT* c.648G > C p.?, was present in the homozygous state in case 1 (Table 4). This change is also predicted to cause aberrant splicing due to the substitution at the last nucleotide position of exon 5.

The final novel change was a missense variant, c.941 T > G p.(Ile314Ser), which was identified in a heterozygous state in case 15 in addition to a heterozygous pathogenic c.748C > T p.(Arg250Ter) nonsense variant; parental testing has not been performed to confirm that these variants are *in trans*. The isoleucine residue at position 314 is highly conserved and multiple lines of computational evidence predict that the substitution of a serine residue at this codon will have a deleterious effect on the *OAT* protein function.

## Discussion

This report describes observational data collated for a cohort of individuals with gyrate atrophy. Hyperornithinaemia and changes in the *OAT* gene were present in all study participants. Diagnosis of gyrate atrophy is frequently achieved surprisingly late. In this cohort, the median age at diagnosis was 8 years. This is comparatively earlier than that described in a systematic review [15] where a median age of diagnosis of 13 years was reported amongst 47 patients. Amongst the patients described here, some remained asymptomatic until adolescence and were diagnosed either on a routine eye assessment or through family screening. Notably, most patients were of south-east Asian origins, potentially suggesting an increased incidence within this group; most also had a history of consanguinity.

A multidisciplinary approach is key for the management of individuals with gyrate atrophy. Ophthalmic imaging is an important investigation in this patient group as it can help monitor disease progression. In this cohort, there was a high degree of variability in terms of the frequency of imaging studies. This may be due to several underlying factors including patient disengagement, the impact of the SARS-CoV-2 pandemic or barriers in communication between service departments. No current standardised guidelines exist for disease monitoring using imaging and different centres utilise varying imaging protocols (that include modalities such as fundus autofluorescence, fluorescein angiography [19] and OCT in addition to fundoscopy). Notably, a large proportion of study participants (78%) developed macular oedema and/or pre-senile cataracts in addition to the core manifestations of nyctalopia and peripheral vision deficits. Macular oedema is a known complication of gyrate atrophy [19, 20], and supportive therapy including administration of topical or oral carbonic anhydrase inhibitors, topical non-steroidal anti-inflammatory drops, intravitreal corticosteroids or intravitreal anti-vascular endothelial growth factor agents has been reported to have a favourable short-term impact on retinal structure [15, 20]. However, large-scale studies are lacking and the effect of these treatments on visual function remains uncertain. Pre-senile posterior subcapsular cataracts are common in individuals with gyrate atrophy and early lens surgery is often required. It is noted that among the subgroup of study participants that required cataract surgery (n = 6), 50% of cases had macular oedema; this highlights that the link between prolonged hyperornithinaemia and distinct ophthalmic complications is likely to be complex. Interestingly, the visual acuity of the study participants who restricted dietary protein was better on average than that of those who did not observe any protein limitation. Although this difference may be due to confounding factors or change, this observation is in keeping with reported evidence that dietary manipulation may correlate with better visual outcomes [7–10, 15–17]. Despite this, progressive visual loss was observed in all patients, highlighting that current management strategies remain suboptimal.

A variety of non-ophthalmologic manifestations was noted in this cohort. The most common finding, observed in one third of patients, was developmental/cognitive impairment. Although learning difficulties have been reported in the literature as a frequently observed patient finding in association with systemic hyperornithinaemia and secondary creatine deficiency [1, 2], our current understanding of the expression of gyrate atrophy related neurocognitive phenotypes is limited. There is similarly lacking evidence pertaining to any correlation between the severity of these manifestations and the levels of raised plasma ornithine and their genotype. Early studies by Kaiser-Kupfer and colleagues [19] reported abnormalities in electroencephalographic testing in individuals with gyrate atrophy, although these did not appear to correlate with any clinical neurocognitive dysfunction. As above stated, the majority of patients in this cohort were born to consanguineous parents, raising the possibility of other genetic factors affecting neurodevelopment.

Similarly, fat free mass biopsy in individuals with gyrate atrophy may reveal abnormalities due to the metabolic effects of raised ornithine and secondary creatine deficiency [1, 15, 21]. However, not all patients with gyrate atrophy exhibit muscular dysfunction [15]. When assessed in clinic, 9 of 11 patients in this cohort had body composition analysis by SECA mBCA Multi-frequency Bioelectrical Impedence Analysis, which indicated a reduced fat free mass [22]. These patients commenced therapy with creatine monohydrate (1.5 mg four times per day) in an attempt to increase fat free mass and muscle strength [23]. Reports of reduced fat free and/or muscle mass in patients with gyrate atrophy need comparison with findings of reduced fat-free mass, by gold standard methodology, in young adults with a range of inherited disorders of protein metabolism as protein restriction alone may be contributory [24].

Given the rarity of the condition, robust data on the management and long-term outcomes in gyrate atrophy are limited. Further, as with other rare genetic disorders, obtaining data on which evidential management can be based remains challenging. Most of the evidence-base for current management strategies involves studies from the 1980s and 1990s [7–10, 15, 17]. No previous randomised control trials or large cohort studies have been described in the biomedical literature [15]. Looking forward, multicentre, collaborative research is required to evaluate available imaging modalities and to develop diagnostic and monitoring recommendations (guidelines) for the long-term surveillance, intervention and treatment outcomes. Furthermore, there is a clinical need for better referral diagnostic pathways to ensure that early dietary modification is offered to newly confirmed cases.

Our findings support the proposal that dietary manipulation may reduce plasma ornithine (although a statistically significant change was not detected in this small-scale series) [16, 17] and that this in turn may confer positive outcomes in terms of limiting vision loss. Furthermore, those individuals diagnosed at an earlier age had lower baseline plasma ornithine levels compared to those diagnosed later. What is often understated however is that the recommended protein restriction imposes a strict and life-long limitation and that patient compliance has been historically poor (less than 70% in this study) due to the poor palatability of dietetic products [17]. Studies investigating the quality-of-life impact of current management strategies for gyrate atrophy are lacking and a balanced assessment of the patient impact of such approaches would help guide future management. In addition to patient challenges with dietary restriction, frequent hospital attendance for appointments with ophthalmologists, metabolic medicine physicians and dieticians can be problematic. Notably, the fact that most affected individuals do not perceive the clinical impact of reducing protein intake and taking supplements/vitamins, can lead to poor engagement and poor compliance. As a result, patient loss to follow up is frequent in later stages of the disease. This limits the capacity for detailed longitudinal studies to obtain further understanding of the disease course. A multidisciplinary approach with a formalised retinal imaging programme is recommended and additional support when transitioning from paediatric to adult services is required. The earlier the low protein diet is introduced, the better compliance in adolescence and adulthood is expected to be. It is noted that participants with learning difficulties who were fully dependent on their parents/carers seemed to follow stricter diet and their compliance with amino acid products was found to be much better.

In conclusion, this report expands knowledge of gyrate atrophy. The restriction of dietary protein reduces plasma ornithine but compliance with treatment can be challenging and a personalised approach is required. Despite the maxim of gyrate atrophy being a “treatable” condition, management remains suboptimal due to poor patient compliance and lack of an effective treatment. Future research into therapies for gyrate atrophy, including novel gene-based interventions such as those utilised in other inherited retinal disorders, would be of significant interest and has the potential to revolutionise clinical management.

### Supplementary Information

**Additional file 1**: **Table S1**: Molecular diagnosis, clinical and biochemical findings in a cohort of 18 patients with gyrate atrophy. **Table S2**: Comorbidities observed in 13 individuals with gyrate atrophy. [Body composition measured in an outpatient clinic setting using a SECA mBCA Bio-Impedance Analysis Machine and completed as per manufacturer’s instructions]. **Figure S1**: Clinical imaging findings from one patient with gyrate atrophy. **Figure S2**: Clinical images (fundus autofluorescence and retinal OCT) from two unrelated patients of similar ages with markedly distinct phenotypes. **Table S3**: Biochemical and visual outcomes at most recent clinic visit using current management strategies. **Table S4**: Genetic Variants and in silico analysis.

## Data Availability

All data generated or analysed during this study are included in this published article.
