# Supplementary material for: Clinical, biochemical and molecular analysis in a cohort of individuals with gyrate atrophy
Source: Orphanet J Rare Dis. 2023 Sep 4;18:265. doi: 10.1186/s13023-023-02840-0 (PMC10476330; doi:10.1186/s13023-023-02840-0)
Supplement: Supplementary file 1 — Additional file 1: Table S1: Molecular diagnosis, clinical and biochemical findings in a cohort of 18 patients with gyrate atrophy. Table S2: Comorbidities observed in 13 individuals with gyrate atrophy. [Body composition measured in an outpatient clinic setting using a SECA mBCA Bio-Impedance Analysis Machine and completed as per manufacturer’s instructions]. Figure S1: Clinical imaging findings from one patient with gyrate atrophy. Figure S2: Clinical images (fundus autofluorescence and retinal OCT) from two unrelated patients of similar ages with markedly distinct phenotypes. Table S3: Biochemical and visual outcomes at most recent clinic visit using current management strategies. Table S4: Genetic Variants and in silico analysis. [file 13023_2023_2840_MOESM1_ESM.docx]

**Additional file 1 - Table 1: Molecular diagnosis, clinical and biochemical findings in a cohort of 18 patients with gyrate atrophy**

| **Patient No.** | **Gender** | **Age Range at Last Ophthalmic Examination (years)** | **Visual Acuity**  **(LogMAR)** | | **Presenting Ophthalmic Symptom(s)**  **(Age at Diagnosis [years])** | **Presence of Macular Oedema (Y/N)** | **Presence of Cataracts (Y/N)** | **Plasma Ornithine Levels at Diagnosis (μmol/L)**  **[NR: 40-150 µmol/L]** | **Average Plasma Ornithine in Last 5 Years**  **(μmol/L)** | **Average Plasma Lysine in Last 5 Years (μmol/L)**  **[NR: 100-160 μmol/L]** | **Genetic Diagnosis, Pathogenic/Likely Pathogenic Variant** |
| --- | --- | --- | --- | --- | --- | --- | --- | --- | --- | --- | --- |
|  |  |  | **Right eye** | **Left eye** |  |  |  |  |  |  |  |
| 1 | M | 31-35 | 0.4 | 1.0 | Increasing myopia (14) | Y | Y | 1120 | 1067 | 87 | c.520+1G>A (homozygous) |
| 2 | M | 21-25 | 0.68 | 0.54 | Increasing myopia (9) | Y | Y | 1018 | 1342 | 73 | c.520+1G>A (homozygous) |
| 3 | F | 26-30 | 0.22 | 0.24 | Increasing myopia (11) | N | Y | 1007 | 863 | 72 | c.520+1G>A (homozygous) |
| 4 | M | 15-20 | 0.32 | 0.44 | Asymptomatic (2) | Y | N | 458 | 868 | 84 | p.(Arg398Ter) (homozygous) |
| 5 | F | 26-30 | 0.42 | 0.3 | Asymptomatic (5) | Y | Y | 695 | 564 | 87 | p.(Arg398Ter) (homozygous) |
| 6 | F | 21-25 | 0.34 | 0.34 | Increasing myopia (6) | N | Y | 1244 | N/A | N/A | p.(Arg398Ter) (homozygous) |
| 7 | F | 5-14 | 0.1 | 0.1 | Increasing myopia (8) | N | N | 576 | 528 | 150 | p.(Pro241Leu) (homozygous) |
| 8 | M | 5-14 | 0.38 | 0.3 | Asymptomatic (4) | N | N | 754 | 597 | 130 | p.(Pro241Leu) (homozygous) |
| 9 | F | 21-25 | 0.5 | 0.64 | Nyctalopia, Reduced peripheral vision (8) | Y | N | 775 | 640 | 106 | p.(Pro241Leu) (homozygous) |
| 10 | F | 31-35 | 0.94 | 0.9 | Increasing myopia, Nyctalopia, Reduced peripheral vision (7) | N | Y | 826 | 572 | 76 | p.(Pro241Leu) (homozygous) |
| 11 | F | 26-30 | 0.5 | 0.6 | Increasing myopia, Nyctalopia, Cataracts (26) | Y | Y | 800 | 718 | 204 | p.(Pro241Leu) (homozygous) |
| 12 | F | 26-30 | 0.3 | 0.4 | Increasing myopia, Nyctalopia (14) | N | Y | 734 | 641 | 103 | p.(Gly51Asp) (homozygous) |
| 13 | M | 21-25 | 0.16 | 0.3 | Asymptomatic (0.83) | N | Y | 458 | 392 | 169 | p.(Arg398Ter)  (homozygous) |
| 14 | M | 0-4 | 0.0 | 0.0 | Asymptomatic (1) | N | N | 1232 | 879 | 195 | p.(Pro241Leu); (heterozygous) p.(Gly353Asp) (heterozygous) |
| 15 | F | 21-25 | 0.22 | 0.4 | Increasing myopia (14) | N | Y | 742 | 716 | 108 | p.(Arg250Ter);  (heterozygous)  p.(Ile314Ser)  (heterozygous) |
| 16 | F | 41-45 | 0.0 | -0.1 | Increasing myopia, Nyctalopia, Cataracts (33) | N | Y | 917 | N/A | N/A | p.(Tyr209Ter); (heterozygous) p.(Pro417Leu) (heterozygous) |
| 17 | M | 15-20 | 0.22 | 0.5 | Increasing myopia, Floaters (14) | N | N | N/A | 458 | 247 | p.(Leu403Pro); (heterozygous) (Leu337ArgfsTer2) (heterozygous) |
| 18 | M | 26-30 | 0.48 | 0.6 | Asymptomatic (5) | N | Y | 920 | N/A | N/A | p.(Pro300LeufsTer13) (homozygous) |

**NB: Two patients for whom genetic data only were available have been excluded from the above table and information regarding their genetic diagnosis is included in Table 4.**

**Y = yes, N = no**

**Additional file 2 - Table 2: Comorbidities observed in 13 individuals with gyrate atrophy**

| **Organ System** | **Comorbidity** | **Number of patients affected** |
| --- | --- | --- |
| Psychological/Neurological | Developmental delay | 6 |
|  | Depression | 2 |
|  | Psychosis | 1 |
|  | Anorexia nervosa | 1 |
|  | Dyslexia | 1 |
|  | Dyspraxia | 1 |
|  | Spastic dysplegia | 1 |
|  | Reduced fat-free mass | 9 |
|  | | |
| Haematological | Anaemia (unspecified) | 1 |
|  | Congenital dyserythropoeitic anaemia | 1 |
|  | Red cell degeneration | 1 |
|  | | |
| Respiratory | Asthma | 2 |
|  | Tuberculosis | 1 |
|  | Vocal cord nodules | 1 |
|  | | |
| Cardiovascular | Congenital heart disease | 1 |
|  |  |  |
| Endocrinological | Precocious puberty | 1 |
|  | Diabetes insipidus | 1 |
|  |  |  |
| Urological | Dysfunctional bladder syndrome | 1 |

**Additional file 3 - Figure 1: Clinical imaging findings from one patient with gyrate atrophy**


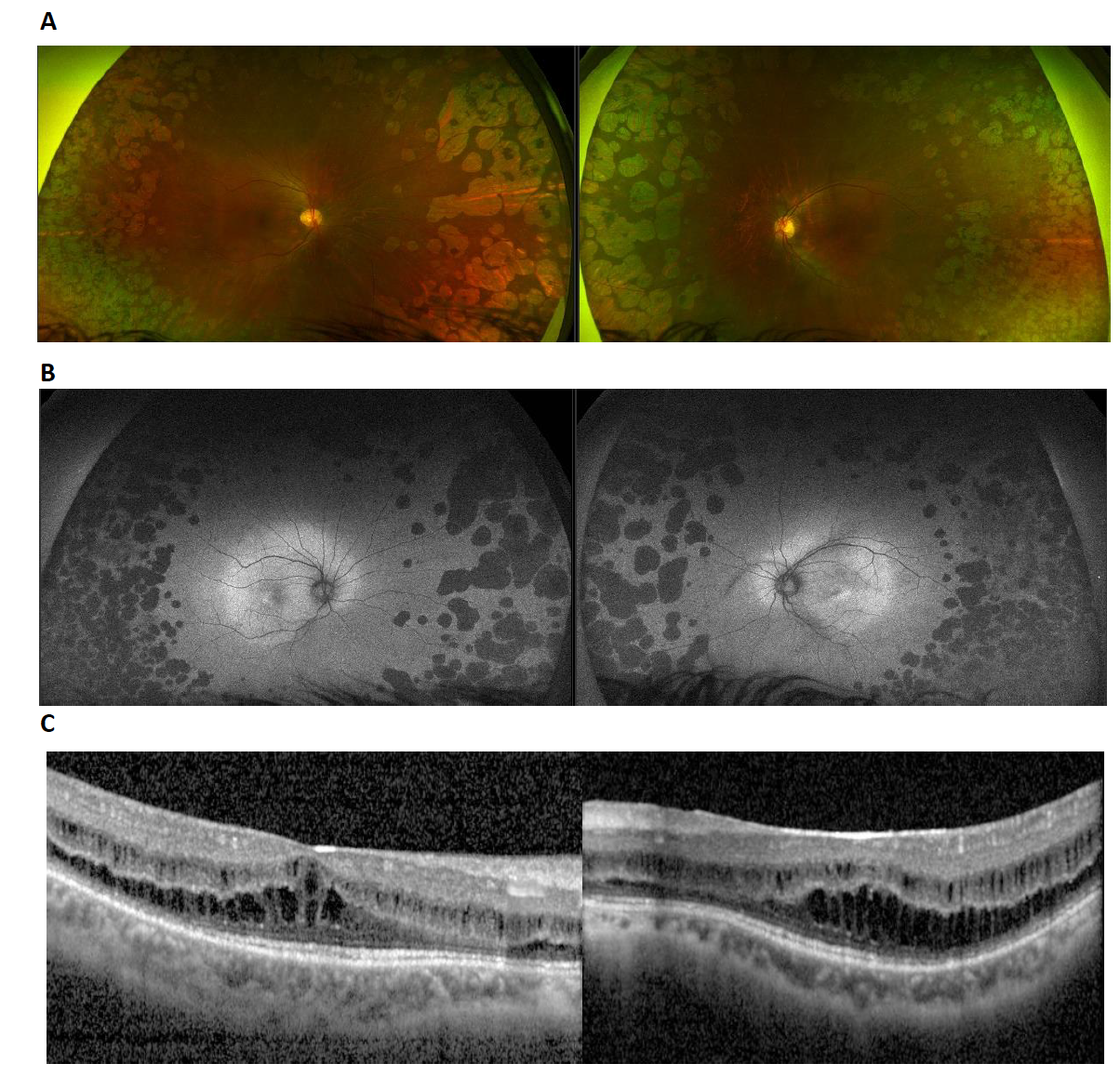


*“Figure 1: Clinical imaging findings from a 22-year-old patient with gyrate atrophy (case 2).* ***A****: Colour fundus images demonstrating characteristic bilateral scalloped, peripheral lesions;* ***B****: Fundus autofluorescence (FA) imaging demonstrating patchy hypo-autofluorescent lesions peripherally in keeping with RPE atrophy;* ***C:*** *Optical coherence tomography (OCT) images of the macula demonstrating bilateral cystoid macular oedema, a known complication of gyrate atrophy.”*

**Additional file 4 – Figure 2: Clinical images (fundus autofluorescence and retinal OCT) from two unrelated patients of similar ages with markedly distinct phenotype**


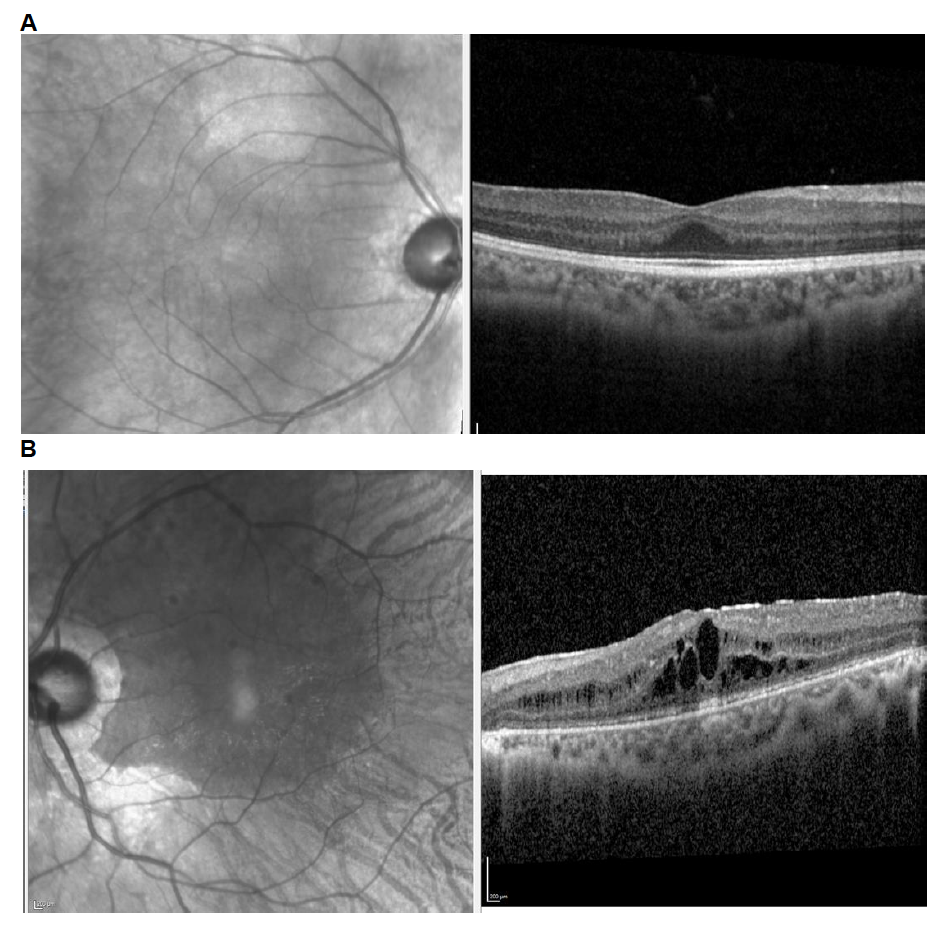


Figure 2: Infrared reflectance and OCT images from two unrelated study subjects with gyrate atrophy. Images from the right eye of case 13 (at age 23 years; A) and from the left eye of case (at 22 years; B) are shown. Case 13 was diagnosed in infancy following an initial presentation with hyperammonaemia, followed a strict dietary protein. restriction from time of diagnosis and had what can be described as an unusually mild phenotype (visual acuity 0.16 LogMAR right and 0.3 LogMAR left). Case 2 was 9 years of age at time of diagnosis, was non-compliant with the recommended diet and went on to develop ophthalmic sequelae including cataract and cystoid macular oedema formation (visual acuity 0.68 LogMAR right and 0.54 LogMAR left). The relevant genetic diagnoses are shown in Table 4.

**Additional file 5 - Table 3: Biochemical and visual outcomes at most recent clinic visit using current management strategies**

| **Patient no.** | **Compliance with current treatment (Y/N/P)§** | **Protein restriction (daily intake [g/kg] excluding EAA)** | **Daily Lysine Supplementation (g)** | **Current additional amino acid supplement regime with total daily dosage (where available)** | **Plasma ornithine at diagnosis (NR: 40-150 μmol/L)** | **Average plasma ornithine in last 5 years (μmol/L)** | **Average plasma lysine in last 5 years**  **(NR: 100-160 μmol/L)** | **Average visual acuity at last ophthalmic examination (logMAR)** |
| --- | --- | --- | --- | --- | --- | --- | --- | --- |
| 1 | P | No restriction | 5 | None | 1120 | 1067 | 87 | 0.7 |
| 2 | N | No restriction | 0 | None | 1018 | 1342 | 73 | 0.61 |
| 3 | P | 0.5-0.6 | 0 | None | 1007 | 863 | 72 | 0.23 |
| 4 | Y | 1 | 4 | EAA (3-4 sachets) | 458 | 868 | 84 | 0.38 |
| 5 | P | No restriction | 4 | EAA (3-4 sachets) | 695 | 564 | 87 | 0.36 |
| 7 | Y | 0.5-0.8 | 0 | EAA (4 sachets)  UCD Amino 5 (1 sachet)  Dialamine (100g) | 576 | 528 | 150 | 0.1 |
| 8 | Y | 0.2-0.3 | 0 | EAA (4 sachets) | 754 | 597 | 130 | 0.34 |
| 9 | Y | 1 | 4 | None | 775 | 640 | 106 | 0.57 |
| 10 | Y | 0.7-1 | 10 | None | 826 | 572 | 76 | 0.92 |
| 11 | P | No restriction |  | EAA  Pyridoxine (300mg) | 800 | 718 | 204 | 0.55 |
| 12 | P | No restriction | 10 | EAA (4 sachets) | 734 | 641 | 103 | 0.35 |
| 13 | Y | 0.15 | 0 | Dialamine (22 scoops)  Phlexyvits  Zinc biotin | 458 | 392 | 169 | 0.23 |
| 14 | Y | 0.4 | 0 | EAA (3 sachets)  Docomega | 1232 | 879 | 195 | 0.0 |
| 15 | N | No restriction | 4 | None | 742 | 716 | 108 | 0.31 |
| 17 | Y | 0.5-0.6 | 0 | Dialamine (100g)  Fruityvit | **+** | 458 | 247 | 0.36 |

**Cases 7, 8 and 14 are paediatric patients; cases 6, 16 and 18 had no available recorded data on ornithine or lysine levels.**

**§ Y/P/N: Yes = compliant with both protein restriction, Partial = compliant with supplementation but not protein restriction, N = not compliant. Compliance was determined by patient metabolic medicine/dietician appointments**

**+ Data not available**

**Additional file 6 - Table 4: Genetic Variants and *in silico* analysis**

| **HGVS description (NM_000274.4)** | **REVEL score** | **GnomAD (v2.1.1)**  Number of non-ref alleles / total number of alleles | **Reference**  (Clinvar & HGMD) |
| --- | --- | --- | --- |
| c.520+1G>A | n/a | Absent | Not reported on HGMD |
| c.461G>A p.(Arg154His) | 0.929 | 2/251370 | Ghosh et al. 2017 |
| c.1192C>T p.(Arg398Ter) | n/a | 2/250954 | Michaud et al. 1995 |
| c.722C>T p.(Pro241Leu) | 0.8999 | 9/251486 | Brody et al. 1992 |
| c.152G>A p.(Gly51Asp) | 0.875 | 3/251488 | Sergouniotis et al. 2012 |
| c.648G>C p.? | n/a | Absent | Not reported on HGMD |
| c.899delC p.(Pro300LeufsTer13) | n/a | Absent | Patel et al. 2018 |
| c.1058 G>A p.(Gly353Asp) | 0.9219 | 10/250560 | Brody et al. 1992 |
| c.748C>T p.(Arg250Ter) | n/a | 2/251456 | Sergouniotis et al. 2012 |
| c.941T>G p.(Ile314Ser) | 0.9169 | Absent | Not reported on HGMD |
| c.627T>A p.(Tyr209Ter) | n/a | 6/282864 | Mashima et al. 1992 |
| c.1250C>T p.(Pro417Leu) | 0.8889 | 8/282378 | Brody et al. 1992 |
| c.1208T>C p.(Leu403Pro) | 0.9449 | 2/282564 | Not reported on HGMD |
| c.1009dup p.(Leu337ArgfsTer2) | n/a | Absent | Not reported on HGMD |

Ghosh et al. <https://pubmed.ncbi.nlm.nih.gov/28468868/>

Michaud et al. <https://pubmed.ncbi.nlm.nih.gov/1612597/>

Brody et al. <https://pubmed.ncbi.nlm.nih.gov/1737786/>

Sergouniotis et al. <https://pubmed.ncbi.nlm.nih.gov/22182799/>

Patel et al. <https://pubmed.ncbi.nlm.nih.gov/30054919/>

Mashima et al. <https://pubmed.ncbi.nlm.nih.gov/1609808/>
